# *Datura metel* stramonium exacerbates behavioral deficits, medial prefrontal cortex, and hippocampal neurotoxicity in mice via redox imbalance

**DOI:** 10.1186/s42826-023-00162-7

**Published:** 2023-06-28

**Authors:** Vincent Onoriode Igben, Wilson Josiah Iju, Omogbiya Adrian Itivere, John Chukwuma Oyem, Peter Sunday Akpulu, Efe Endurance Ahama

**Affiliations:** 1 Department of Human Anatomy, Delta State University, Abraka, Nigeria; 2 Department of Pharmacology and Therapeutics, Delta State University, Abraka, Nigeria; 3 Department of Human Anatomy, Novena University Ogume, Delta State, Nigeria; 4 Department of Human Anatomy, Ahmadu Bello University, Zaria, Nigeria

**Keywords:** Anxiety, Cognitive deficits, *Datura metel*, Depression, Neurotoxicity, Neurodegeneration, Oxidative stress

## Abstract

**Background:**

*Datura metel* (DM) stramonium is a medicinal plant often abused by Nigerians due to its psychostimulatory properties. Hallucinations, confusion, agitation, aggressiveness, anxiety, and restlessness are reported amongst DM users. Earlier studies suggest that DM induces neurotoxicity and affect brain physiology. However, the exact neurological effects of DM extract in the medial prefrontal cortex (mPFC) and hippocampal morphology have not been elucidated. In this study, we evaluated the hypothesis that oral exposure to DM extract exerts a neurotoxic effect by increasing oxidative stress in the mPFC and the hippocampus and induces behavioral deficits in mice.

**Results:**

DM methanolic extract exposure significantly increased MDA and NO levels and reduced SOD, GSH, GPx and CAT activities in mice brains. In addition, our results showed that DM exposure produced cognitive deficits, anxiety, and depressive-like behaviour in mice following oral exposure for 28 days. Moreover, the mPFC and hippocampus showed neurodegenerative features, loss of dendritic and axonal arborization, a dose-dependent decrease in neuronal cell bodies’ length, width, area, and perimeter, and a dose-dependent increase in the distance between neuronal cell bodies.

**Conclusions:**

Oral exposure to DM in mice induces behavioural deficits, mPFC and hippocampal neuronal degenerations via redox imbalance in the brain of mice. These observations confirm the neurotoxicity of DM extracts and raises concerns on the safety and potential adverse effects of DM in humans.

**Graphical abstract:**

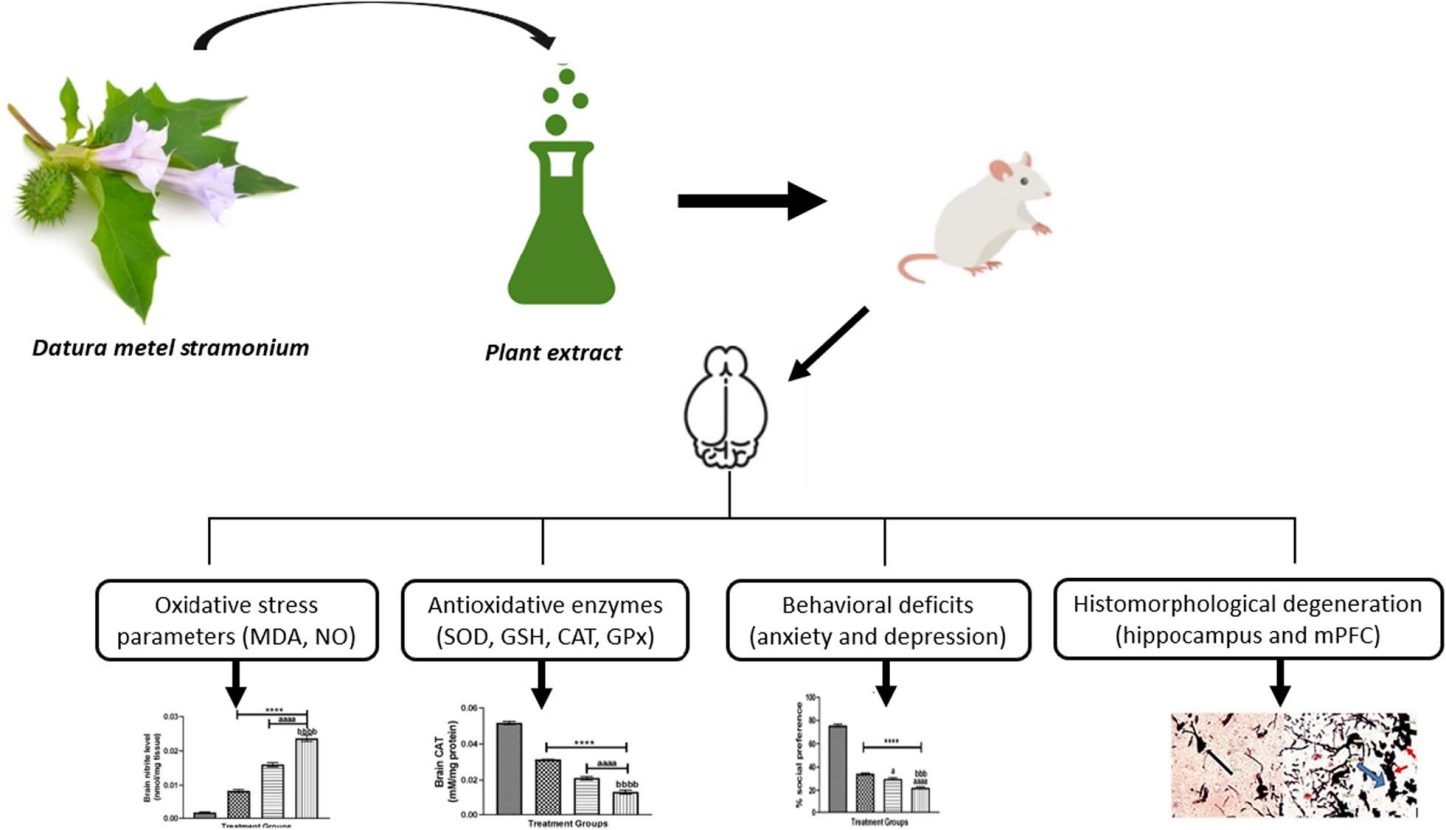

## Highlights

- DM increased oxidative stress in the brain.
- DM depleted antioxidant defence status in mice.
- DM administration led to cognitive deficits.
- DM exposure led to depressive and anxiety-like behaviour.
- DM affected mPFC and hippocampal cell morphology and morphometry.

## Background

According to the World Health Organization (WHO), about 21,000 medicinal plants or supplements are used to treat different diseases [1, 2]. Nevertheless, some plants are often abused due to their hallucinogenic potential. *Datura metel* (DM) falls into this category.In Nigeria, young people abuse its psychostimulant properties for mood-altering benefits [3]. *Datura metel stramonium* is a plant that belongs to the family of Solanaceae with over 85 genera and approximately 2,800 species worldwide [4]. This plant is widely distributed in Nigeria, and it is known locally by different ethnic groups- Igbo myaramuo, Hausa – Zakani, Yoruba – gegemu [5]. Moreover, in folklore medicine, DM is employed to treat various pathologies due to its antimicrobial, antioxidant, anti-inflammatory, antipyretic, and analgesic properties [6–9]. Its leaves, seeds, and fruits are added to liquor or are dried, rolled, and smoked like cigarettes [10, 11]. Its numerous properties and uses are attributed to its phytochemical constituents.

DM poisoning and toxicity have been recorded in both human and animal studies [5, 11, 12]. In humans, hallucinations, restlessness, and heavy fall were reported among Fulani youth in Nigeria after consuming DM seeds [13], while confusion, agitation, mydriasis, and hallucination were reported in children who consumed DM seeds in Nigeria [5, 14–17]. In animal studies, most researchers have demonstrated that exposure to DM induces organ toxicity [18–21]. The toxic compounds in DM are belladonna alkaloids, including atropine, scopolamine and hyoscyamine [22]. These alkaloids traverse the blood–brain barrier through muscarinic receptors [23]. In ascending order, the alkaloid content of DM is present in the root, leaves, seeds/fruits, and stems and is greatest in the flowers [24].

Until now, most studies conducted on DM are case studies, while other studies focus on its phytochemistry and medicinal properties. Few studies have proven its effects on the brain in animal models [11, 25–27]. We acknowledge the research conducted by [28], which focused on the role of datumetine, an active ingredient of DM, on hippocampal NMDAR activity. The current study stands out in its approach by simulating real-life human consumption, taking into account the impact of the phytoconstituents in the entire leaf of DM as a whole and not in parts. Aside from studying the overall effect of DM leaves on the brain, another distinct advantage of our approach is that it combines biochemical, neurobehavioral, stereological and histological techniques to unveil the neurotoxic effects of DM. Consequently, we have limited knowledge of the neurotoxic potentials of DM and how it affects cognition. With this background, understanding the effect of graded doses of DM in the hippocampus and mPFC in mice will provide evidence of the neurological implications of its abuse. We analysed the behavior of experimental animals following oral exposure to DM for 28 days using different batteries of behavioral assessment tests for spatial and non-spatial memory, anxiety, and depression-like behavior. In addition, we analysed the oxidative stress parameters and antioxidant enzymes in mice brains treated with DM. Furthermore, we investigated its effect on the histoarchitecture of the mPFC and hippocampus using histological and stereological techniques.

## Results

### *Datura metel* leaf extract administration increases brain lipid peroxidation and depletes antioxidant status

**Fig. 1:**
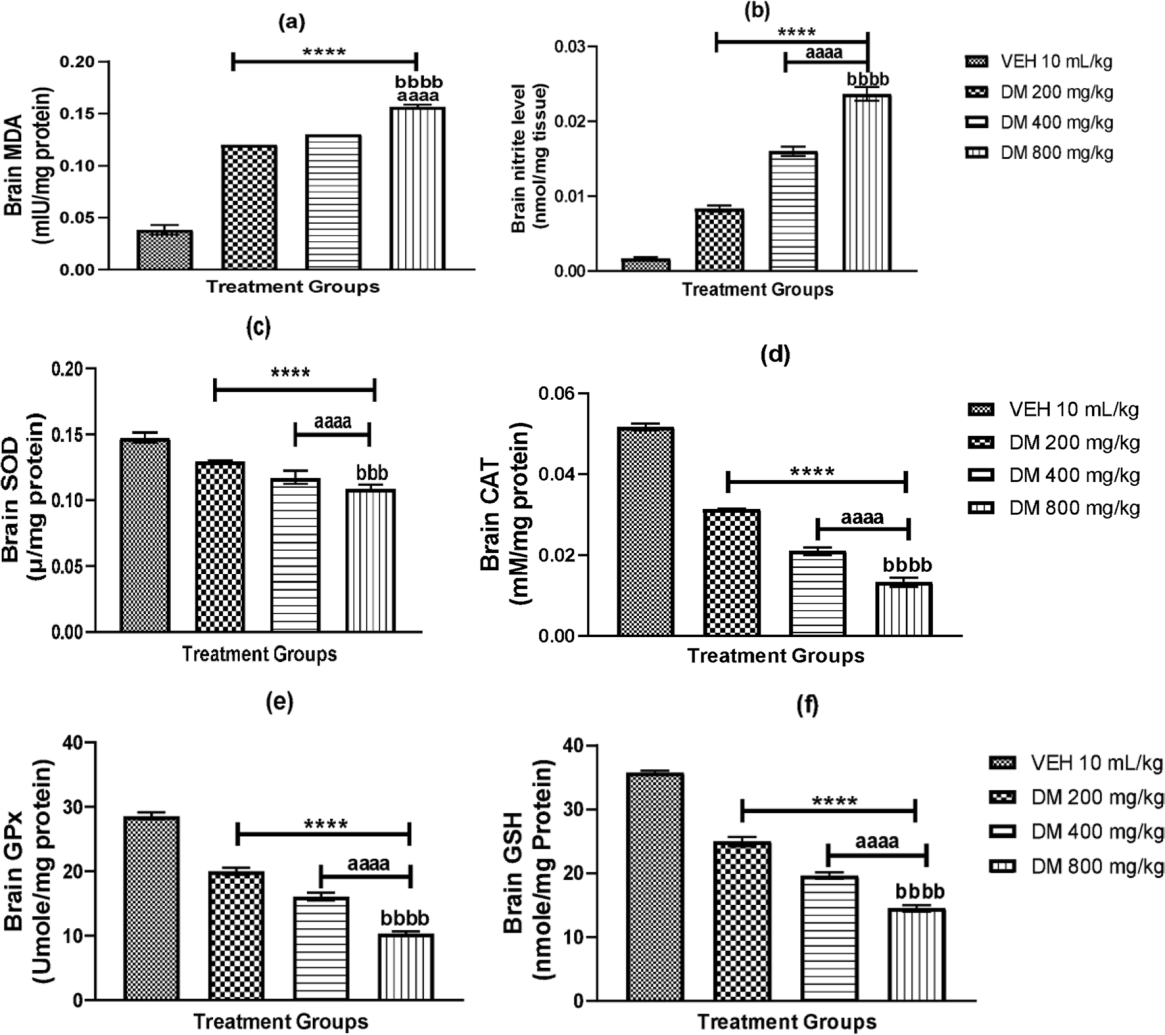
Effect of *Datura metel* leaf extract on brain oxide-nitrosative markers and enzymatic antioxidants in mice: Malondialdehyde, MDA **a**, Nitrite (NO) **b**, superoxide dismutase, SOD **c**, catalase, CAT **d**, glutathione, GSH **e** and glutathione peroxidase, GPx **f** Bars represent the mean ± S.E.M (*n* = 6). One-way ANOVA followed by Bonferroni’s posthoc test revealed significant differences between various treatment groups. ****Denotes *p* < 0.0001 as compared to the control group. ^aaaa^Denotes *p* < 0.0001 as compared to DM (200 mg/kg) group. ^bbbb^denotes *p* < 0.0001 as compared to DM (400 mg/kg) group. *VEH* Vehicle, *DM* *Datura metel*

Biochemical assay analysis for markers of oxidative and antioxidant activities showed dose-dependent significant changes in MDA, SOD, NO, CAT, GPx and GSH. Between-group comparisons revealed a significant increase in MDA and NO levels in the brains (*p* < 0.0001) of *DM* mice (Fig. 1a, b) with a dose-dependent decrease in brain concentrations of SOD, CAT, GPx and GSH (*p* < 0.0001), compared to the control groups (Fig. 1c–f).

### *DM* leaf extract diminishes memory performance in experimental animals

**Fig. 2:**
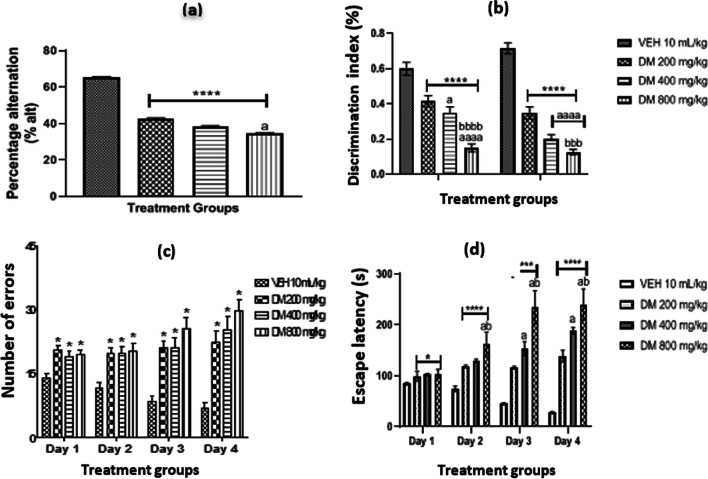
Effect of *Datura metel* leaf extract on memory as measured by the percentage (%) alternation using Y maze test (YMT) **a**, discrimination index using novel object recognition test (NORT) **b**, number of errors **c**, and escape latency using Barnes maze test (BMT) **d**, are presented in Fig. 2 Value represents the mean ± S.E.M of Six animals/group. One-way ANOVA revealed that there are significant differences between various treatment groups. *, **** denotes *p* < 0.05, 0.0001 when compared to control group; ^a, aaaa^ denotes *p* < 0.05, 0.0001 compared to DM 200 mg/kg; ^bbb, bbbb^ denotes *p* < 0.001, 0.0001 compared to DM 400 mg/kg. *VEH* Vehicle, *DM* *Datura metel*

The effect of *DM* administration on memory as measured by the percentage (%) alternation behaviour using the Y maze test (YMT), discrimination index using the novel object recognition test (NORT), the number of errors and escape latency using the Barnes maze test (BMT) are presented in (Fig. 2). Comparison between groups showed a dose-dependent decrease (*p* < 0.0001) in the percentage of alternation in all experiment groups when compared with the control (Fig. 2a). In the NORT (Fig. 2b), *DM* administration at a graded dose led to a significant dose-dependent decline (*p* < 0.0001) in the discrimination index of the experimental animals in both 4 h and 24 h assessments using the NORT when compared with the control. Furthermore, in the Barnes maze, oral administration of *DM* significantly increased the number of errors and escaped latency in all experimental groups compared with the control (*p* < 0.05, 0.001, 0.0001) (Fig. 2c & d). In general, the maximal effect of *DM* was observed in group D, which received the highest dose of the extract.

### *DM leaf extract* induces anxiety-like behavior in experimental mice

**Fig. 3:**
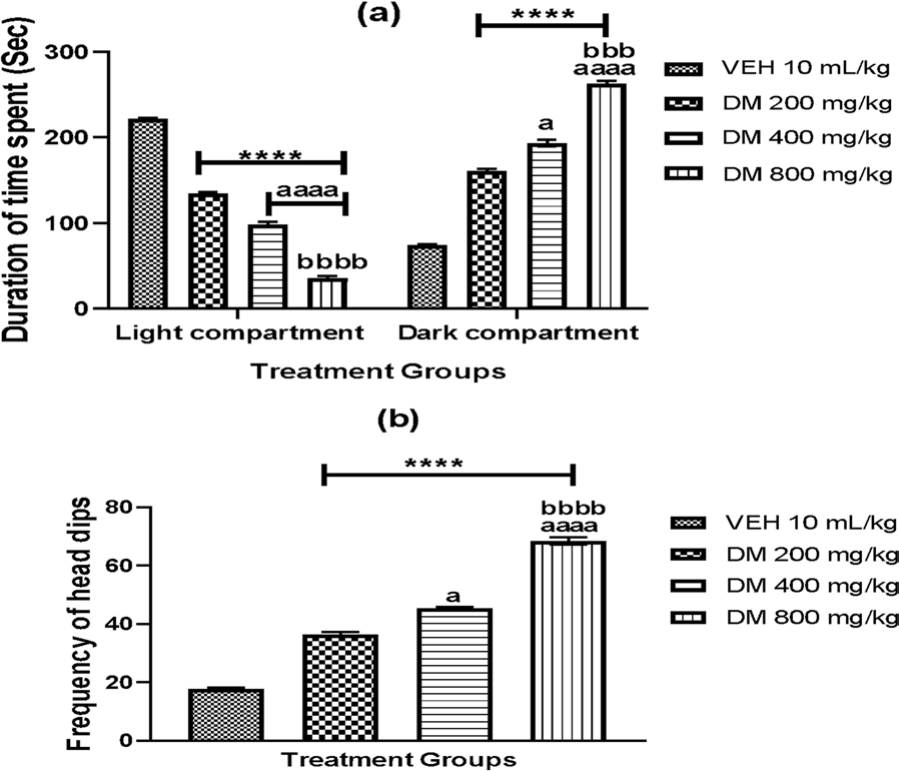
The effect of *Datura metel* leafextracts on anxiety-like behavior as measured by the frequency of entry and time spent in the dark chamber using the light/dark transition box test **a** and frequency of head dip using the hole board test (HBT) **b**. The value represents the mean ± S.E.M of Six animals. One way ANOVA revealed that there are significant differences between various treatment groups for frequency of entry and time spentand frequency of head dip **** denotes *p* < 0.0001 when compared to the control group; ^a aaaa^ denotes *p* < 0.05, 0.0001 compared to DM 200 mg/kg; ^bbb, bbbb^ denotes *p* < 0.001, 0.0001 compared to DM 400 mg/kg. *VEH* Vehicle, *DM* *Datura metel*

Anxiety-like behavior in mice was assessed using the percentage index of open-arm avoidance in the elevated plus-maze, frequency of entry and time spent in the light and dark chambers of the light/dark transition box test and frequency of head dip in the hole board test. Oral administration of *DM* led to anxiety-like behaviour in mice with a dose-dependent increase in the index of open-arm avoidance compared to the controls (*p* < 0.05) (Table 1). Moreover, DM-treated mice showed anxiety-like behavior by spending less time and more time in the light and dark compartments of the light/dark box, and this difference in duration was highly significant and dose-dependent (Fig. 3a). To confirm this anxiety effect induced by DM, mice were assessed using the hole board test. Again, DM at graded doses led to a significant increase (*p* < 0.0001) in the frequency of head dips in the experimental group when compared with the control groups (Fig. 3b).

**Table 1:** Effect of *Datura metel* leaf extract on anxiety-like behaviour in mice using elevated plus maze (EPM)

Treatment	Open arm entry	Open arm duration	% Open arm entry	% Open arm duration	Index of open arm avoidance
VEH10mL/kg	5.83 ± 0.48	73.17 ± 3.57	42.33 ± 2.99	24.39 ± 1.19	66.50 ± 1.29
DM 200 mg/kg	3.50 ± 0.43*	41.50 ± 3.13*	26.63 ± 2.23	13.83 ± 1.04*	79.61 ± 1.36*
DM400 mg/kg	2.83 ± 0.31*^a^	28.67 ± 2.40*a	21.47 ± 1.96*a	9.557 ± 0.80*^a^	84.49 ± 0.84*^a^
DM 800 mg/kg	1.67 ± 0.33*^ab^	17.00 ± 2.28*ab	14.85 1.87*ab	5.665 ± 0.76*^ab^	88.91 ± 1.32*^ab^

Values are expressed as Mean ± SEM (*n* = 6) (One-way ANOVA followed by Bonferroni post hoc test). *, a, b *p* < 0.05 considered statistically significant when compared with the Control, *Datura metel* extract (200 mg/kg) and DM (400 mg/kg) treated groups, respectively *VEH* Vehicle, *DM* *Datura metel*

### *DM* leaf extract exacerbates depressive-like behavior in mice

**Fig. 4:**
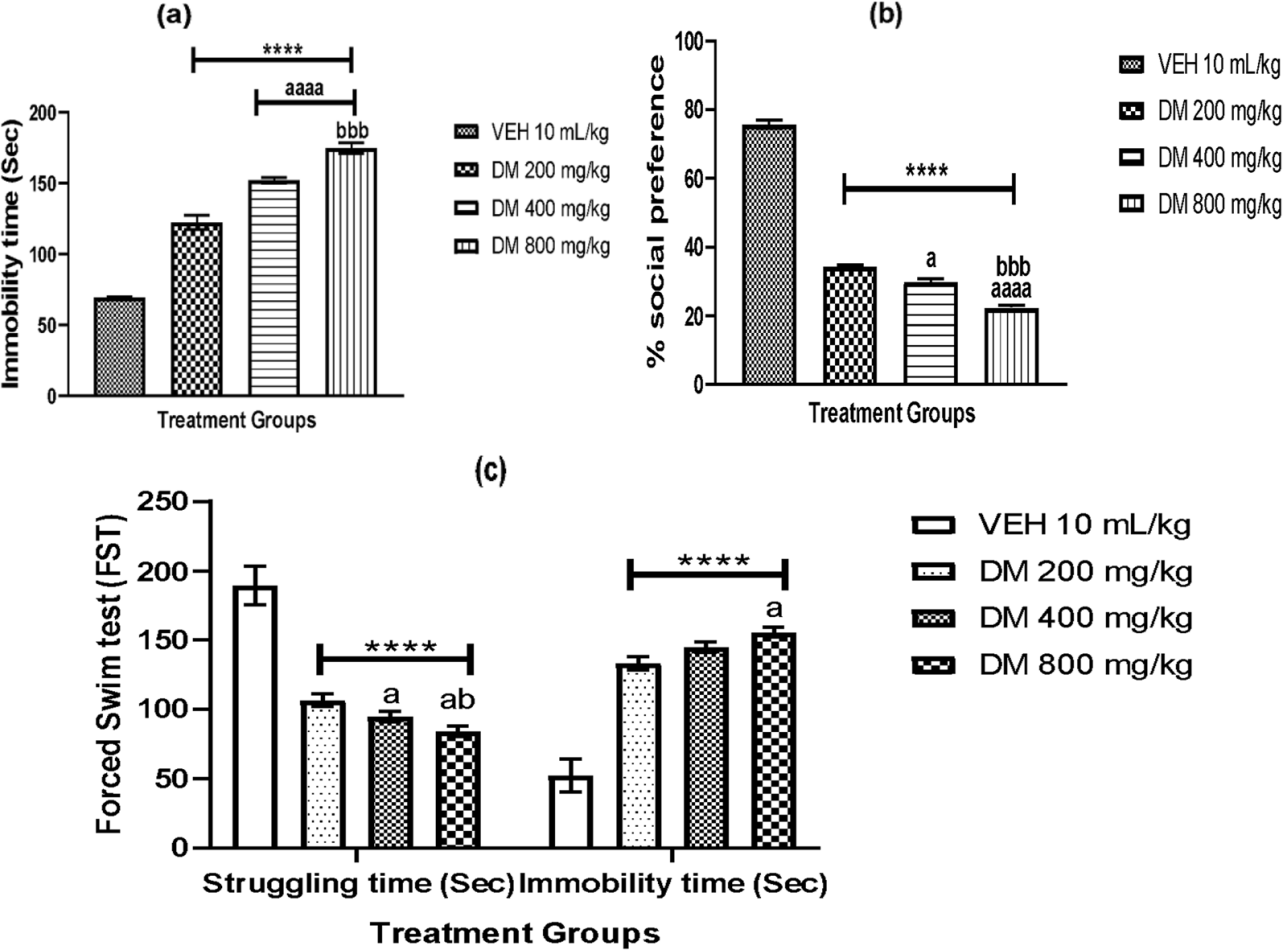
Effect of *Datura metel* leaf extract on depressive-like behavior as measured by the immobility time using tail suspension test (TST) **a** sociability index using social interaction chamber (SIT) **b** and Forced swim test **c** Value represents the mean ± S.E.M of Six animals/group. One-way ANOVA revealed that there are significant differences between various treatment groups. **** denotes *p* < 0.0001 when compared to the control group; ^a and aaaa^ denotes *p* < 0.05, 0.0001 compared to DM 200 mg/kg; ^bbb^ denotes *p* < 0.001 compared to DM 400 mg/kg. *VEH* Vehicle, *DM* *Datura metel*

The effect of *DM* leaf extract on depressive-like behaviors as measured by the immobility time, percentage social preference and struggling time against immobility in the tail suspension test, social interaction test, and forced swim test are shown in Fig. 4. One-way ANOVA revealed a dose-dependent increase (*p* < 0.0001) in the total number of times the animals were immobile (Fig. 4a). This immobility time was higher in the experimental groups and highest in group D mice which received the highest dose. However, experimental mice that were administered graded doses of *DM* showed a dose-dependent decrease (*p* < 0.0001) in the percentage of social preference compared with the control groups (Fig. 4b). In addition, *DM* caused a significant dose-dependent decrease in the struggling time and a dose-dependent significant increase (*p* < 0.0001) in the immobility time in the treated groups compared to the control group in the force swim test, thus confirming the hypothesis that *DM* induces depressive-like behavior (Fig. 4c).

### Effect of *DM* leaf extract on mice hippocampal histology.

**Fig. 5:**
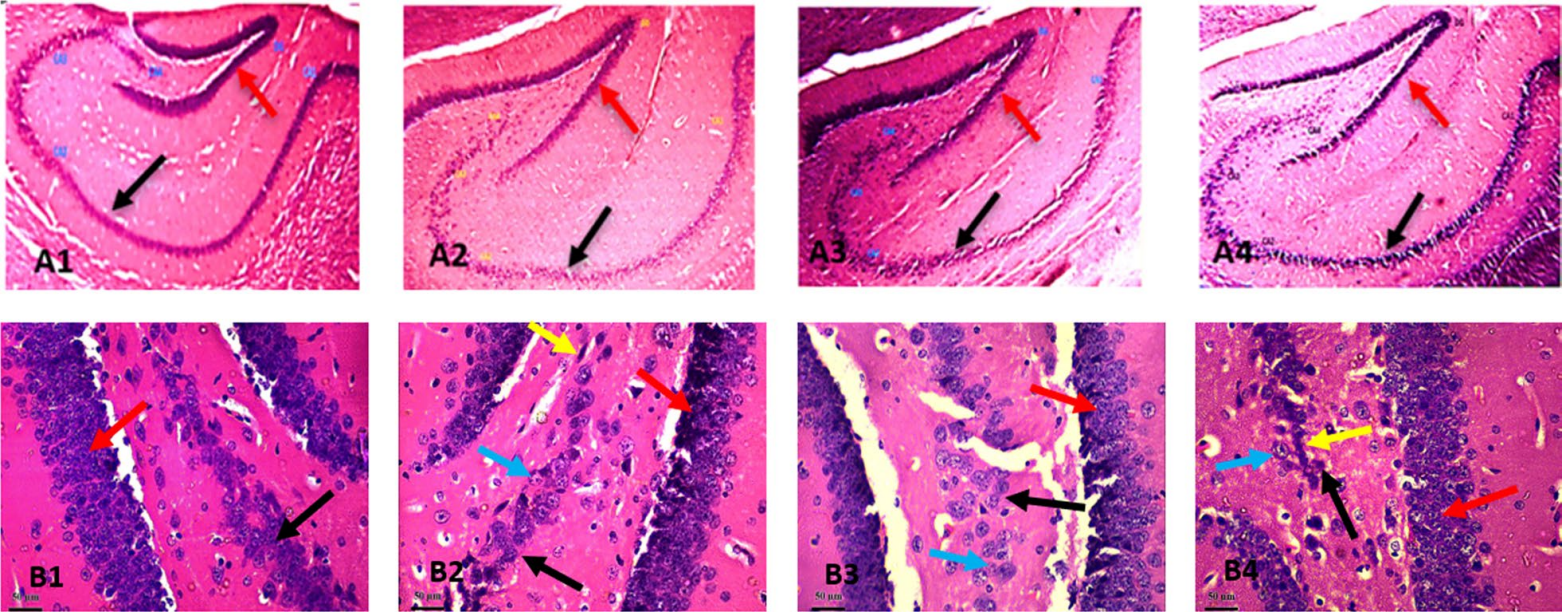
Representative photomicrographs of the effect of *Datura metel* leaf extract on pyramidal cells of the hippocampus. **A**: VEH; **B**: DM (200 mg/kg); **C**: DM (400 mg/kg); **D**: DM (800 mg/kg);Plate A1–B4: Black arrow– shows sparsely packed pyramidal cells in the treated groups compared with control, red arrow: – shows sparsely packed granule cells in the treated groups compared with control plate B1–B4: Black Arrow– pyramidal cells, red arrow—granule cells; yellow arrow – nuclear pyknosis, blue arrow- cell body chromatolysis. H and E stain, Mag: × 40, × 250; Calibration bar for all figures = 0.01 mm (10 µm). *VEH* Vehicle, *DM* *Datura metel*

The effect of *DM* leaf extract on the hippocampus is presented in Figs. 5a and b. H&E brain slides (Fig. 5) showed sparsely packed pyramidal cell layer with degenerative changes in all mice treated with *DM* extract compared with the control mice, which showed normal histoarchitecture of the hippocampal pyramidal cells. Histoarchitectural details of the treated groups showed very few neuronal cell bodies with characteristic features of pyknosis and chromatolysis.

**Fig. 6:**
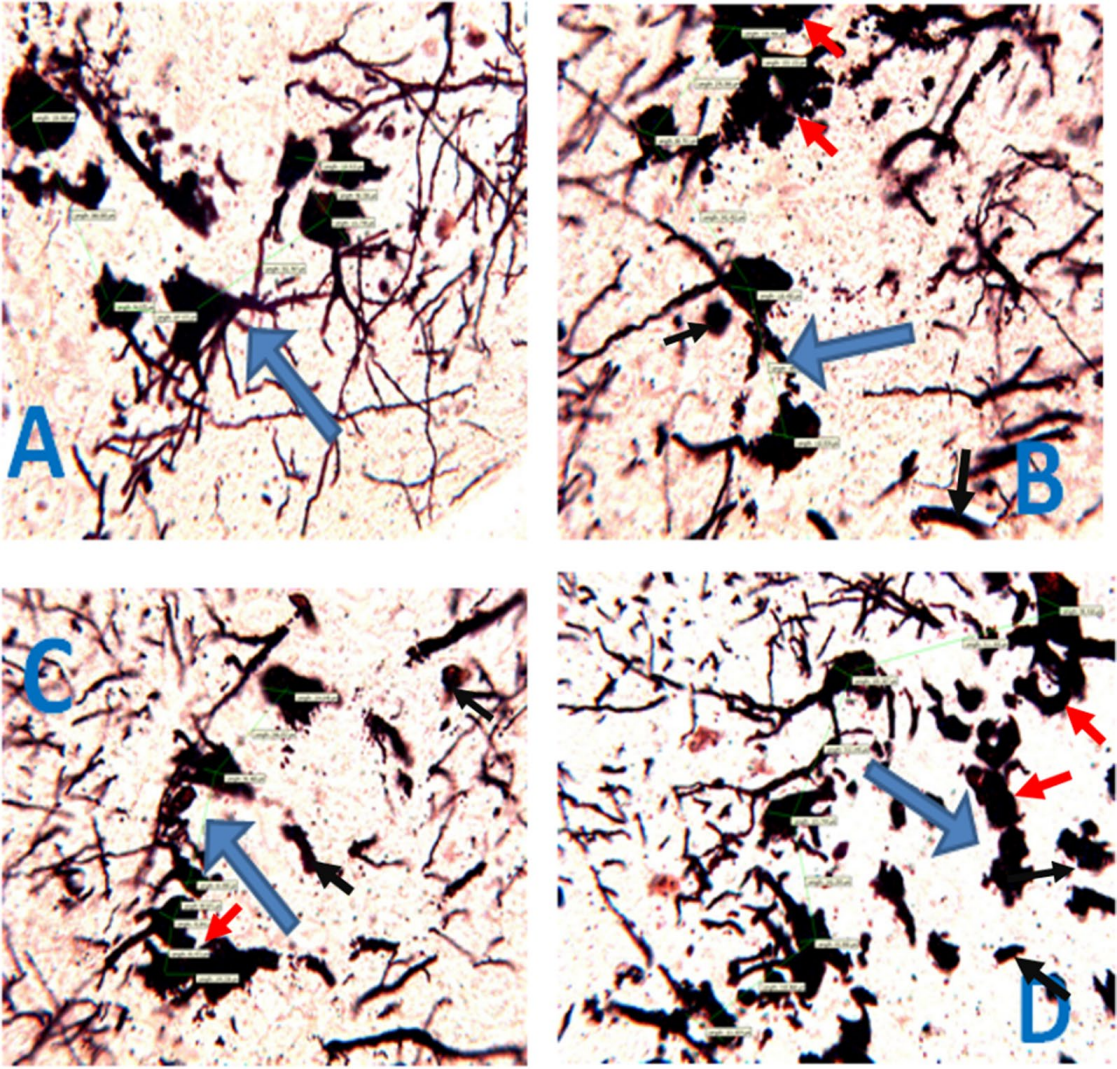
Representative photomicrographs of the effect of *Datura metel* on pyramidal cells of the hippocampus treated with *Datura metel* leaf extract. **A**: VEH; **B**: DM (200 mg/kg); **C**: DM (400 mg/kg); **D**: DM (800 mg/kg); Blue Arrow– pyramidal nerve axons, red arrow – pyknotic clumped cell bodies, black arrow – necrotic cell bodies. (Golgi) stain; Mag: × 400; Calibration bar for all figures = 0.01 mm (10 µm) *VEH* Vehicle, *DM* *Datura metel*

On the Golgi stain photomicrograph (Fig. 6a–d), mice treated with *DM* showed significant distortions of the stroma cells and loss of dendritic arborizations of the pyramidal cells compared to the control group. The morphological features of the cell bodies in the treated groups showed irregularly sized cell bodies with smaller diameters compared to the control. The treated groups also showed pyknotic features, clumping cell bodies, loss of nerve cell branches as well as necrotic cell bodies compared to the control. These histological features are in keeping with degenerative changes and neuronal cell death.

### Effects of *DM* on the cellular parameter of hippocampal pyramidal cells in mice

**Fig. 7:**
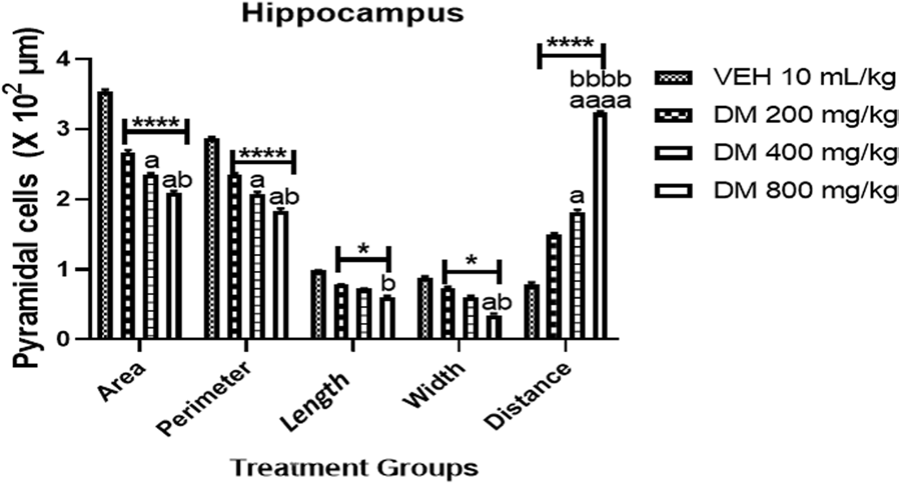
Effects of *Datura metel* leaf extract on hippocampal pyramidal cell morphology. Bars represent the mean ± S.E.M (*n* = 6). One way ANOVA followed by Bonferroni’s posthoc test revealed significant differences between various treatment groups. ****Denotes *p* < 0.0001 as compared to the control group. ^a, aaaa^Denotes *p* < 0.05, 0.0001 as compared to DM (200 mg/kg) group. ^b, bbbb^Denotes *p* < 0.05, 0.0001 as compared to DM (400 mg/kg) group. *VEH* Vehicle, *DM* *Datura metel*

The effect of *DM* leaf extract on hippocampal pyramidal cell morphology is presented in Fig. 7. As seen in all treated groups, *DM* administration led to a significant (*p* < 0.0001) dose-dependent increase in the intercellular distance of the pyramidal cell with a corresponding significant dose-dependent decrease in area, length, width and perimeter of the hippocampal pyramidal cell as compared to control group. Conversely, mice that received *DM* at the highest dose showed a higher increase in the intercellular distance of the pyramidal cell with the corresponding decreased area, length, width and perimeter of the hippocampal pyramidal cell compared to the control group.

### The effect of *DM *leaf extract on neuronal cells of mPFC in mice

**Fig. 8:**
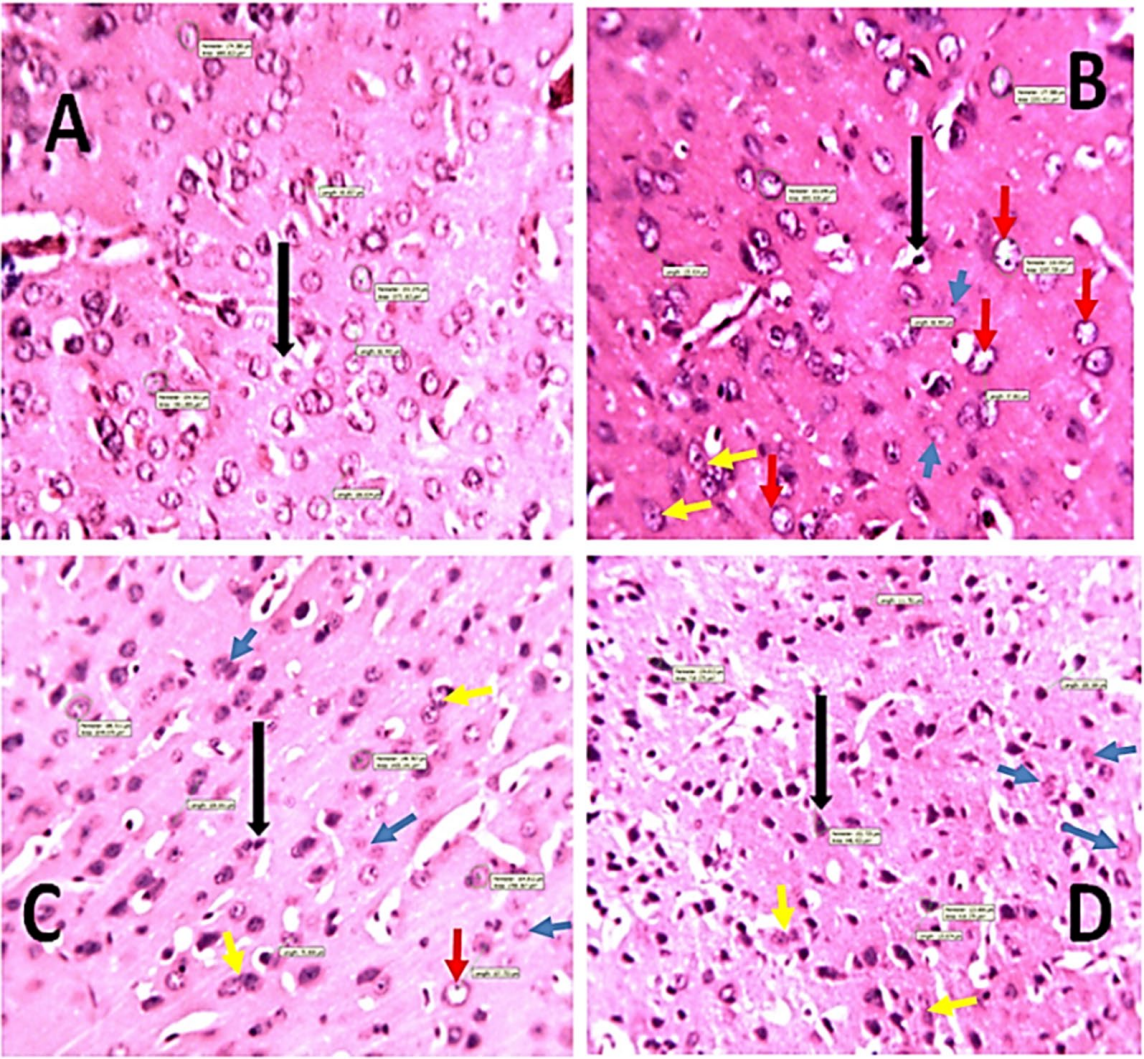
Representative photomicrographs of the effect of *Datura metel* leaf extract on the medial prefrontal cortex neuronal cells. **A**: VEH; **B**: DM (200 mg/kg); **C**: DM (400 mg/kg); **D**: DM (800 mg/kg); Black Arrow– shows shrinking nerve cells in the treated groups compared with control, red arrow – neuronal vacuolation, blue arrow- cell body chromatolysis, yellow arrow – nuclear pyknosis. (H and E) stain; Mag: × 250; Calibration bar for all figures = 0.01 mm (10 µm) *VEH* Vehicle, *DM* *Datura metel*

**Fig. 9:**
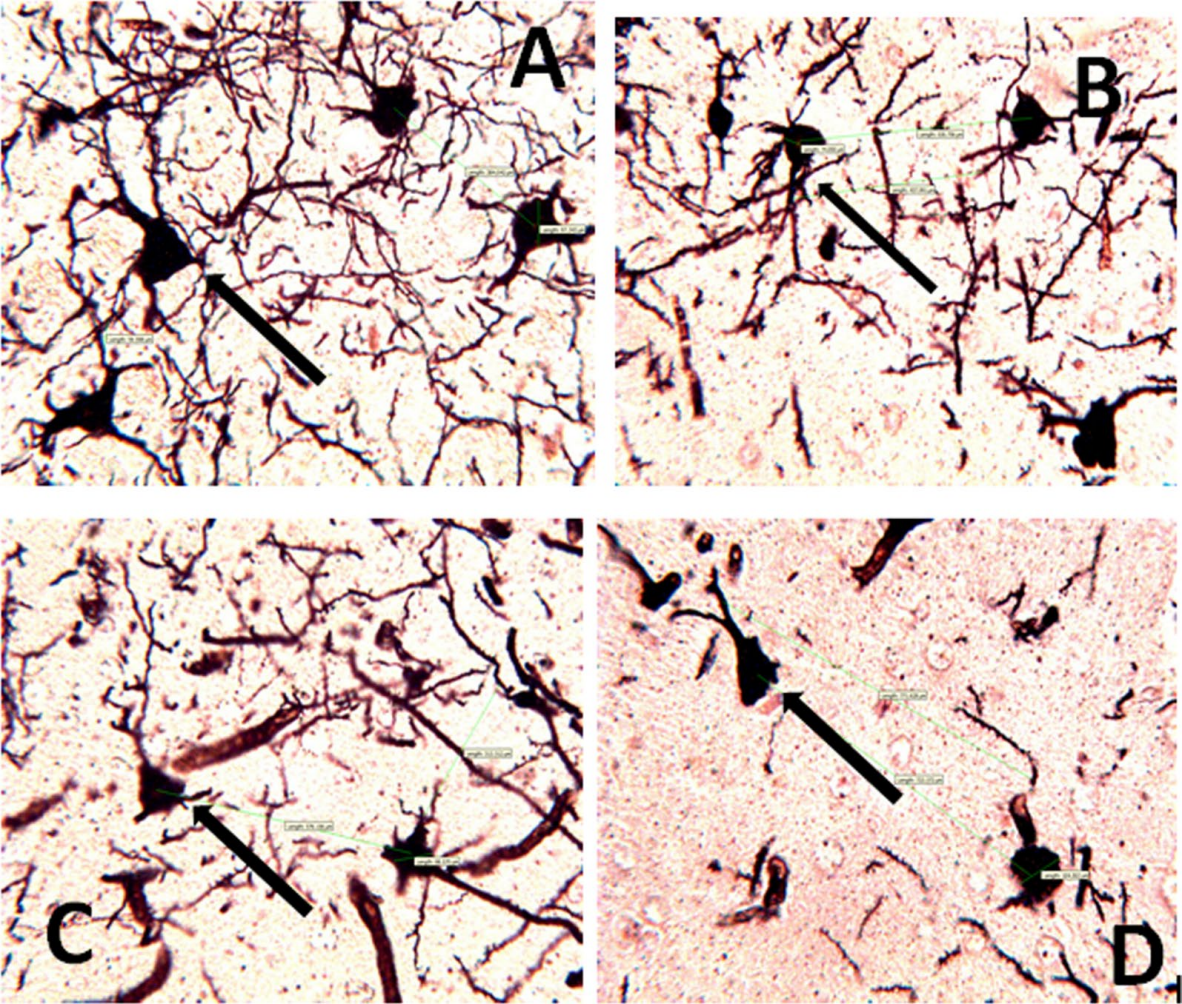
Representative photomicrographs showing the effect of *Datura metel* leaf extract on the axons of the medial-prefrontal cortex at graded dose. **A**: Control (deionized water); **B**: 200 mg/kg of DM; **C**: 400 mg/kg); **D**: 800 mg/kg. The black arrow shows nerve cells with dendrites. (Golgi × 250); Mag: × 400; Calibration bar for all figures = 0.01 mm (10 µm) *VEH* Vehicle, *DM* *Datura metel*

The effect of *DM* leaf extract on mPFC histology stained with H&E (Fig. 8) and Golgi stain (Fig. 9) is shown below. The control H&E stained mPFC showed normal abundant granular cells characterised by eosinophilic cytoplasm and peripherally placed nucleoli in dispersed neuropil. mPFC of the DM-treated groups shows distorted nerve cell soma with neuronal vacuolations. mPFC cell bodies in treated mice also showed shrinkage in a halo spaced neuropil together with nuclear pyknosis and cell body chromatolysis when compared with the control group. On the other hand, the Golgi stain photomicrograph (Plate 9a–d) showed distortion of the nerve cell morphology with loss of axons, nerve cell soma and dendritic arborizations. These features were remarkable in group D (800 mg/kg). The above features are suggestive of neuronal degeneration.

### Effects of *DM* leaf extract on the cellular parameters of mPFC in mice

**Fig. 10:**
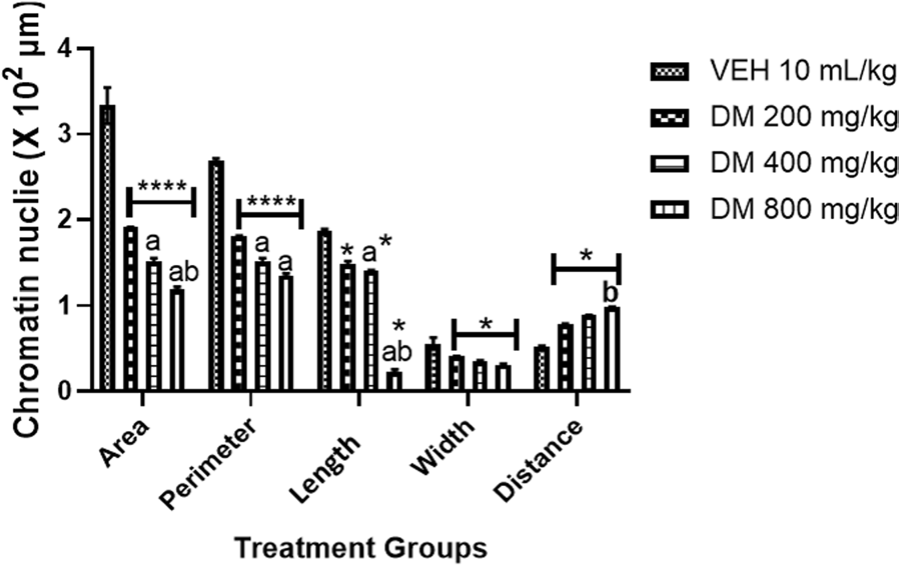
Effects of *Datura metel* leaf extract on mPFC morphology. Bars represent the mean ± S.E.M (*n* = 6). One way ANOVA followed by Bonferroni’s posthoc test revealed significant differences between various treatment groups. ****Denotes *p* < 0.0001 as compared to the control group. ^a, aa, aaaa^Denotes *p* < 0.05, 0.01, 0.0001 as compared to DM (200 mg/kg) group.^bbbb^Denotes *p* < 0.0001 compared to DM (400 mg/kg) group. *VEH* Vehicle, *DM* *Datura metel*

The effects of *DM extract* on the cellular parameter of the medial prefrontal cortex in mice is presented in Fig. 10. *DM* administration led to a dose-dependent increase (*p* < 0.0001) in the intercellular distance and dose-dependent decreases in the length of the dendritic cell body, nerve cell area, perimeter and width compared to the control group. A heightened effect of *DM* was observed in group D animals, which received the highest dose of *DM*.

## Discussion

In this study, we investigated the effects of oral administration of graded doses of *DM* methanolic extract on memory and cognition in a behavioral context and the neurotoxic potentials of *DM* in the mPFC and hippocampus of mice. Regarding the neurotoxicity potential of *DM*, we observed that oral administration of *DM* induced neuronal degeneration in the hippocampus and mPFC with cellular hypoplasia and loss of dendritic arborizations. In addition, we observed a significant dose-dependent increase in oxidative stress and depletion of the antioxidant status of all treated experimental mice. Remarkably, this suggestive neurotoxicity and increased oxidative stress in mice were reflected in the behavioral assessment of the animals, which revealed memory impairment and cognitive deficits, anxiety and depressive behaviors in mice following oral administration of *DM*.

### *Datura metel* leaf extract administration increases brain lipid peroxidation nitrosative activities and depletes antioxidant status

The increased levels of oxidative and nitrosative substances play a role in multiple brain pathologies. Lipid peroxidation, due to cellular excitotoxicity, results from a rapid rise in free radical generation either through cyclooxygenase or nitric oxide synthases [29, 30]. Furthermore, free radicals’accumulation in the hippocampus and prefrontal cortex leads to memory deficits via alteration in long-term potentiation [31, 32].

In the present study, oral administration of *DM* extract led to a dose-dependent increase in the levels of MDA and NO in mice. MDA is a biomarker of lipid peroxidation, which has high reactivity and toxicity, resulting from sustained oxidative stress production [33]. These increase in MDA levels, observed in mice administered different *DM* doses, indicate lipid peroxidation. In addition, our study showed a significant dose-dependent increase in nitric oxide concentration in the brain of *DM* mice compared to the controls. NO, synthesised by the enzymatic actions of nitric oxide synthetase (NOS), is expressed in neurons, glia and vascular cells. It functions as a retrograde synaptic neurotransmitter, neuronal intracellular signalling molecule, neuromodulation and synaptogenesis [34].

Conversely, in pathological conditions, NO reacts with superoxide anion to form peroxynitrite, which diffuses through the membranes of neurons and damages neuronal biomolecules, facilitating early progressions of neurodegenerations. According to [35], peroxynitrite is crucial for the nitration of proteins and oxidation, lipid peroxidation, mitochondrial alternations, and cell death and an increase in the brain concentrations of NO can initiate neurodegeneration [36]. Our finding indicates that DM administration increased NO levels in the brain, leading to the neurodegenerative changes observed in hippocampal and medial prefrontal cortex histology. The increased level of NO observed in our study could be linked to the activities of NOS, which are triggered by oxidative stress. Our finding implies that DM might induce brain oxidative damage by catalysing biological macromolecule peroxidation in the brain, resulting in cellular proliferation, cell injury and cell death. This finding is in tandem with a study that reported a significant increase in MDA levels in rats’ liver, kidney, heart, and brain following *Datura stramonium* seed extract [37].

Equally important are our findings from the biochemical analysis of antioxidative enzymes. These enzymes are triggered to combat oxidative damage, abrogate ROS generation, and terminate the actions of already generated radicals. The first line of the enzymatic antioxidant defence system includes SOD, CAT and GPx [38]. Antioxidant enzymes metabolise ROS generated from lipid peroxidation into less toxic molecules; for example, SOD which is essential for converting superoxide radicals to hydrogen peroxide (H2O2), halts oxidative stress by interacting with other neuroprotective antioxidants [39–42]. In this study, we assayed the levels of SOD, CAT, GPx and GSH following oral administration of DM for 28 days. Our findings indicated that oral administration of DM extract at low and high doses depleted the antioxidant defence system by inducing a dose-dependent decline in SOD, CAT, GPx and GSH activities compared with the control mice. This DM's depletion of the antioxidant defence system suggests increased oxidative stress productionand is in synchrony with the finding from MDA and NO.

SOD plays a crucial role as the first line of defence by detoxifying superoxide radicals. It converts generated superoxide radical anion to H2O2 and prevents free radical inactivation of dehydratase [43]. The decline in the activities of SOD observed in our study might indicate the brain's vulnerability to neurotoxicity induced by DM. Previous studies have reported a disruption in SOD activities [37] and mutations in SOD-1 and SOD-2, genes encoding SOD in neurodegenerative diseases [44, 45]. Gene mutations in SOD-1 result in mitochondrial malfunction, gene expression alterations, caspase activation, abnormalities in the cytoskeleton and unusual protein pathologies [46].Also, loss or decline in activity of SOD increased aging-related neurological pathologies and reduced life span in mice. In contrast, increased expression of SOD-1 is reported to possess neuroprotective potential against neurotoxicity [47]. This assertion correlates with findings from our study that DM administration might produce neurotoxicity by downregulating the activities of SOD-1 and SOD-2 genes [47].

Another essential antioxidant investigated in this study is GPx. This antioxidant reduces H2O2 to water to compensate for its deleterious effects [48], and this removal inhibits the formation of ROS and hydroxyl radicals formed by the reaction between H2O2 and Fe2 + . GPx is localised in the cytosol, mitochondrial and peroxisomes of neurons and is known to eliminate intracellular peroxides more than catalase [49]. Our study showed a decline in GPx activity following oral administration of DM in the brain of mice, thus indicating a reduction in the brain's ability to combat H2O2 created due to oral administration of DM. Subsequent studies stated that GPx protected against doxorubicin-induced apoptosis [50, 51] and could also protect against cell death in response to cytotoxic drugs, which was contrary to the findings of this study, wherein DM exposure depleted Gpx activities in mice [52].

GSH and CAT are employed in elucidating the actions of xenobiotics on the oxidative profile of animals [53]. GSH plays vital roles in numerous physiological processes, including cell proliferation, sulphur transportation, cell signalling, protein synthesis, phytochelatin synthesis, gene expression and detoxification of xenobiotics [54]. We also analysed the roles of GSH and CAT in DM-exposed mice; again, oral administration of graded doses of DM led to a dose-dependent significant decrease in GSH and CAT activities. This also implied that oral administration of DM decreases GSH and CAT activities, and the proposed mechanism is based on the inability of GSH and CAT to eliminate H2O2 generation induced by DM.

The rapid increase in MDA and NO indicates oxidative stress, while the decline in enzymatic (SOD, GPx, CAT) and non-enzymatic (GSH) also depicts oxidative stress. This increase in oxidative stress could result from an increase in peroxide accumulation in the brains of DM mice leading to the formation of ROS and disruption in the activities of CAT and GPx, the principal antioxidant defence enzymes that eliminate intracellular peroxides. This assertion is supported by previous studies which showed catalase and GSH depletion in cells having increased levels of H2O2 following oxidative damage [55, 56].

### *Datura metel* leaf extract administration diminishes memory performance in experimental animals

This study assessed short-term and long-term memory in mice following oral exposure to DM extract. Short-term memory enables information retrieval for fractions of seconds to several minutes, and it is regulated by neural activations in the prefrontal cortex [57, 58], while long-term memory and other memory types are solely dependent on the neural activations in the hippocampus [59], lesions to these brain areas affect memory consolidation [60]. To confirm whether DM induced cognitive dysfunctions in this study, we assessed the experimental animals' working memory using the Y-maze, NORT and Barnes maze test in mice. We report a dose-dependent decrease in the percentage of alternation in mice administered graded DM doses compared to the control. This low percentage or decreased alternation indicates a poor working memory in DM-exposed mice. We further assessed spatial memory in experimental animals using the Barnes maze test. The Barnes maze test is a hippocampal-dependent exploratory task where animals need to learn about environmental cues and a fixed escape direction [61]. Again, our result showed a triple-dose-dependent increase in errors and escaped latency in the DM groups compared with the control. This increase could be interpreted as the inability of the DM-administered mice to fully comprehend the cues in the maze and make an escape using the fixed escape direction as quickly as possible. These results corroborate with the NORT, another relative and highly effective test for investigating learning and memory in mice; we observed a significant dose-dependent decrease in the memory index of mice exposed to DM extract compared with controls. All these findings indicate that oral DM administration for 28 days altered short-term memory in mice. This observation concords with a recent study that reported memory impairment in mice following the administration of datumine, a potent constituent of DM [28], and maybe attributed to dysregulations of corticosterone, BDNF and CREB by the phytoconstituents of DM, notably datumine as previously reported. Dysregulation in these pathways results in depression and memory deficits [62]. Likewise, the observed effect might also be that the extract decreases the activities of acetylcholine, which is imperative in cognition, but this needs further investigation.

### *Datura metel* leaf extract induces anxiety-like behavior in experimental mice

After 28 days of *DM* administration, findings from EPM revealed that treated mice preferred closed arms, with an increase in the percentage index of open-arm avoidance in treated groups. We also observed immobility, freezing, and defecation in the DM mice on entering the open arms, which are characteristic anxiogenic behaviour and thus asserted that DM may provoke anxiety in the treated mice.We further investigated the anxiogenic potentials of DM using the light/dark transition box and the hole board test. Our findings revealed that DM-administered mice showed a dose-dependent increase in the frequency of head dipping, entry and time spent in the dark chamber and a decrease in the time spent in the light chamber compared with the controls – which also is an indication of fear and anxiety. Studies in animal models have shown that animals administered with anxiogenic substances tend to spend more time in the dark chamber of the light/dark transition box than in the light chamber [63, 64]. Hence, these results confirmed that oral exposure to DM extract for 28 days in rats induces anxiety-like behaviour in mice.

Anxiety-like behaviour reported in the current study may be related to neurotoxicity observed in emotional processing brain structures of the limbic system. The frontal cortical circuitry controls impulses and emotions by inhibiting the emotion centres in the brain and the hippocampus, which downregulates the hypothalamic–pituitary–adrenal axis stress response [65]. Another region that might be susceptible to this toxicity is the central amygdala. Although it was not investigated in the current study, we presume that neuronal degenerations in this region might contribute to this finding because the amygdala receives input from the hippocampus and plays a vital role in the fear and flight response, control of aggression and retrieval of memory/experiences related to stress and anxiety [66]. Our findings might also stem from physiological alterations induced by DM in the activities of certain neurotransmitters, including GABA, glutamate, serotonin, and norepinephrine, which have been implicated in anxiety and mood states [67].

### *Datura metel* leaf extract exacerbates depressive-like behavior in mice

Earlier research reported cognitive deficits as an element of depression and the roles of medicinal plants in improving depression [68, 69]. Thus, we assessed depressive-like behavior in mice following exposure to DM using the TST, social preference test and FST. In line with previous research, we expected an improvement in the experimental animals’ mood, but surprisingly, our findings showed heightened depressive behaviors following oral administration of DM.

The TST is applied in therapeutic medicine to measure the efficacy of antidepressants. We subjected animals to TST after 28 days of administration with DM and measured the immobility time. Our findings revealed that mice administered varying doses of DM had a higher immobility time than the control, and the effect was doubled in the group that received the highest dose of DM. This higher immobility time in mice indicates their unwillingness to carry out the task effectively and hence the higher number of times spent. Similarly, we report a significant dose-dependent decrease in DM mice's percentage social preference test compared to the controls, thus suggesting social deficits, a principal characteristic of depression. In this test, we observed diminished social interest in mice administered DM, indicating that DM impairs social motivation and mood.

In the present study, we also employed FST to assess the effect of stress in mice administered DM extract orally for 28 days. DM produced a dose-dependent decrease in the struggling time and a significant increase in the mobility time. These result patterns indicate that oral exposure to DM at 200 mg/kg, 400 mg/kg and 800 mg/kg exacerbated depressive behavior and did not possess antidepressant properties at the doses administered.

This finding may be ascribed to the increased levels of oxidative stress reported in the current study, especially NO. NO is known to regulate major brain neurotransmitters such as noradrenaline, dopamine, glutamate and serotonin that play vital roles in the neurobiology of depression. The modulatory functions of NO is utilized to produce antidepressants [36, 70], and a decreased level of NO synthesis in the brain induces an antidepressant effect [36, 71]. This study reports an increased NO level in mice administered DM, thus justifying the depressive-like behavior observed in the experimental animals.

### *Datura metel* leaf extract induces neurotoxicity and loss of hippocampal pyramidal cells

The hippocampus forms a significant part of the circuit of Papez, and its neurons project to various cortical and subcortical structures [72]. It plays a crucial role in learning and memory. The primary cell type of the hippocampus is the excitatory pyramidal neuron which incorporates contextual, spatial, and emotional information and relays output to various cortical and subcortical structures in the brain [73]. Here, we investigated the effect of DM on the hippocampus by exposing mice to varying doses of DM and assessed the histoarchitectural details of the hippocampus. Findings from DM-treated mice's hippocampus indicated neuronal degeneration in the hippocampal pyramidal cells. These cells were characterised by loss of cellularity, pyknosis and chromatolysis. We also emphasized neuronal distortion by visualising the entire neuronal morphology and quantifying the cell body diameter, areas, perimeter, length, width, and distance from the cell bodies. For the first time, we report neuronal distortion in the cell bodies of the pyramidal cells with loss of dendritic and axonal arborizations in all treated groups. This loss of dendritic arborizations may impede synaptic transmissions and thus affect memory. This agrees with a previous study that reported retardation in the hippocampus following prenatal exposure to DM leaf ethanolic extract [27].

Furthermore, our hippocampal morphometric analysis showed that oral exposure to DM in mice resulted in a dose-dependent decrease in the area, perimeter length and width of pyramidal cell bodies and a significant dose-dependent increase in the distance between pyramidal cells. These significant changes infer hippocampal cell distortion in mice treated with DM. Therefore, our study suggests that oral exposure to DM confers neurotoxicity, leading to degenerative neuronal changes in the hippocampus, and affecting hippocampal functions and connectivity. This observation is in line with the memory impairment observed in the behavioral assessment of mice.

### *Datura metel* leaf extract induces neurotoxicity and loss of cellularity in the medial prefrontal cortex

The mPFC is responsible for adaptive response, long-term memory, memory consolidation and short-term memory. This region also plays a role in the conditioning and extinction of fear [74]. The current study showed that histological examination of the DM-treated mice revealed significant cytoarchitectural distortions, pyknosis, chromatolysis, vacuolations and shrinkage in the mPFC. The Golgi stain profiling revealed severe loss of cellularity, axons and dendritic arborizations, distorted nerve cell soma with nuclear shrinkage. These characteristic findings maybe hallmarks of brain pathology and mediate neurodegeneration [75].

Moreover, the morphometric analysis revealed a dose-dependent significant decrease in areas, perimeter, length, and width of mPFC neuronal cell bodies of treated mice with a dose-dependent significant increase in the distance between cell bodies. The morphometric analysis further confirms the neurodegenerative changes observed and thus affirms that oral exposure to DM induces neurotoxicity, leading to neuronal degeneration in the mPFC of treated mice [25, 26].

Although the exact mechanism of DM-induced neurotoxicity in the hippocampus and medial prefrontal cortex is still unclear, the current study proposes that DM extract induces brain neurotoxicity through heightened oxidative stress, leading to cognitive dysfunctions in mice. This may be attributed to atropine, scopolamine and hyoscyamine, which are known as the toxic components of DM [22]. These alkaloids inhibit the muscarinic effect of acetylcholine. For example, scopolamine and atropine antagonise acetylcholine at muscarinic receptors [76]. Scopolamine has been shown as a depressant and has the effect of blocking short-term memory [77]. Atropine, on the other hand, alters anticholinergic signalling, which results in changes in heart rate, hyperthermia, sedation, delirium, psychological imbalance, mydriasis, aggressiveness, and memory loss [78]. In addition, a likely mechanism of action for this observed neurotoxicity might be associated with the dysfunctionality of the critical enzymes of purinergic signalling [79] or because of the effect of datumetine on N-methyl-D-aspartate receptors (NMDAR) [28]. However, phytochemical analysis of DM has revealed the presence of other active compounds such as alkaloids, tannins, steroids, saponins, scopolamine, atropine, flavonoids, phenols, and glycosides. Electrolytes such as calcium, magnesium, and iron are found in the seeds, roots, and leaves [4, 8, 10, 80], while other chemical components of this plant include ( +)-pinoresinol-O-β-D-diglucopyranoside, ( +)-pinoresinol-O-β-D-glucopyranoside, p-tyrosol, trans-N-p-coumaroyltyramine, cis-N-p-coumaroyltyramine, 4-hydroxy-N-(4-hydroxyphenethyl) benzamide, phenylethyl beta -D-glucopyranoside, kaempferol-3-O-β-D-glucopyranosyl-(1–2)-β-D-galactopyranosyl-7-O-L-Rhamnopyranoside, and kaempferol-3-L-Rhamnopyranoside [80].

## Conclusions

The current study showed that oral administration of DM induced biochemical, behavioral, and histomorphological alterations in the mPFC and hippocampus in mice and supports our hypothesis that increased oxidative stress induced by *DM* exposure results in cognitive deficits, anxiety, depressive-like behavior and neuronal degenerations in the mPFC and hippocampus. These observed neurotoxic effects of *DM* leaf extract may be linked to its constituent alkaloids. The findings from the current study, together with previous studies, provide evidence-based scientific justification for the regulation and control of the use of this plant.

## Methods

### Animal care

Twenty-four male Albino Swiss mice weighing between 20 and 24 g were obtained from the animal house, Faculty of Basic Medical Sciences, Delta State University Abraka, Nigeria. Animals were housed in well-ventilated plastic cages and maintained under laboratory conditions of humidity (55 ± 10 g/m^3^) and a 12 h light/ dark cycle with controlled temperature (21 ± 1º C). Mice were acclimatised for two weeks, fed with a mice-pelletised diet, and allowed to drink water ad libitum.

### Plant collection and extraction

Fresh leaves of the *DM* plant were collected from a farm in Obinomba, Delta State, Nigeria. They were identified and authenticated by Dr Ekeke Chimezie of the Department of Plant Science and Biotechnology, the University of Port-Harcourt, with Herbarium Number: *UPH/P/281*.

The crude methanolic extract of *DM* leaves was prepared following the methods of Tijani et al. (2015). Freshly harvested *DM leaves* were air-dried to prevent denaturation of the plant’s phytochemical constituents by the sun. The air-dried leaves were pulverised into powdered form with an electric blender. Using the cold maceration technique, 50 g of the powdered leaves (using Mettler weighing balance instrument S/N 754550, Zurich, Switzerland) were dissolved in 500 mL of 70 per cent v/v methanol at a temperature of 25 °C and left for 72 h. Methanol was used because it is the most suitable solvent for plant extraction [81], and often users consume the plant by soaking the dried leaves in alcohol. The resulting mixture was filtered with Whitman filter paper (No.1) and concentrated to dryness using a vacuum rotary evaporator at 40^0^C. A yield of 75 g of dark green hygroscopic paste-like residue was obtained, stored in universal bottles and refrigerated at 4^0^C for subsequent use. The dosage of DM administration was based on results obtained from our pilot and previous studies, which reported LD50 of DM as 328.5 mg/kg/b.wt [10, 82].

### Ethical consideration

All experimental procedures and protocols reported in this study comply with the animal guidelines for the treatment and use of laboratory animals of the National Institute of Health (NIH) and were approved by the Research Ethical Committee of the Faculty of Basic Medical Sciences, Delta State University Abraka, Nigeria with protocol number *REC/FBMS/DELSU/21/83.*

### Experimental design

**Fig. 11:**
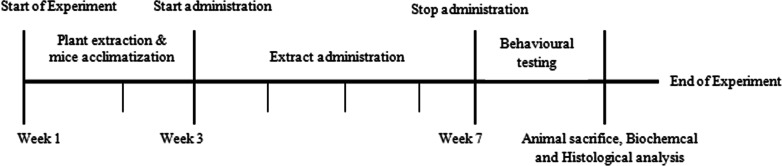
shows the timeline for the experiments. The extract administration began in the 3rd week and ended in the 7th week. In week 5, the animals started behavioural training, while in week 7, they were tested using various behavioural paradigms

After two weeks of acclimatisation, the experimental animals were randomly divided into four groups (*n* = 6). Group 1, the control group, received deionised water, while groups 2, 3, and 4 received 200 mg/kg, 400 mg/kg and 800 mg/kg b. wt of *DM* methanolic extract daily for 28 days, respectively. The plant extract was administered orally between 7:00 am to 10:00 am, using an orogastric tube. The experiment lasted for seven weeks (see Fig. 11).

### Behavioral assessment

All experimental mice were subjected to batteries of behavioral tests to assess spatial working memory, non-spatial working memory, anxiety, and depression. Three different behavioral tests were conducted to rule out all forms of bias and confirm the results obtained from the assessment. Mice from each group were assigned to a particular behavioral test. Behavioral training was carried out three consecutive days in the 5^th^ week, while testing was done in the 7^th^ week after the extract administration. An observer scored the duration and frequency of the behavior blind to the experimental groupings, treatment and expected outcome of the observation.

### Assessment of spatial working memory using Y-maze

The Y-maze model was used to assess spatial memory based on the inherent ability of the mice to explore new environments. The equipment comprises three evenly spaced arms (4.1 m long, 0.5 m wide, 1.5 m high and 120° apart). Mice in each group were put in one of the arm compartments, usually arm A, for consistency, and allowed to explore the maze for 5 min. Arm feedback was graded when the four paws of the animals were entirely in the arms of the Y-maze. An alternation is an entry into all three arms in consecutive order. The percentage alternation, memory index, was recorded by measuring the frequencies of alternations in each arm and was calculated using the formula; the total alternation number/total number of entries-2) × 100. The Y-maze apparatus was carefully cleaned with 70% ethanol between the trials to remove visible urine, fecal matter, and odor cues [83].

### Assessment of spatial working memory using Barnes maze

The Barnes maze was used to test the spatial working memory, using the animal’s natural preference for the dark environment and escape through holes. It consists of a circular platform with perimeter holes (12, 20 or 40 depending on the diameter) with a height of 140 cm. A pre-trial habituation session was performed, and the mice were positioned in the centre of the open maze platform at a random orientation for 5 min. They were allowed to seek the escape route (hole). The number of head dips into the non-target escape hole (errors) during the exploration time and the time taken to look for the escape route (latency) was recorded. After each trial, the maze was cleaned with 70% alcohol to avoid odour cues [61].

### Assessment of non-spatial memory using the Novel Object Recognition (NOR) test

The effect of *DM* on memory performance was assessed using the novel object recognition test (NOR). It consists of an open-field chamber (60 cm × 50 cm × 40 cm) with cylindrical objects of the same dimensions (4.5 cm in diameter and 11.5 cm in height). Each mouse was placed for 5 min on opposite sides of the open-field chamber (8 cm from the walls and 34 cm from each other) in the centre of two identical objects (A and B). After 30 min, object B was replaced with object C, a novel object, new to mice and distinct from each other. Mice were returned to the chamber and were left to explore items A and C for 5 min. After each assessment, the time spent exploring each object in both phases was recorded, and the index of discrimination (a non-spatial memory feature measure) was measured as the difference between the times spent exploring new and familiar objects, divided by the total amount of time spent on both objects [84]. The arena and objects (A, B, C) were carefully cleaned with 70% ethanol before and after assessment.

### Assessment of anxiety levels using elevated plus maze (EPM)

The EPM investigates the anxiolytic and anxiogenic effects of drugs/substances in animal models. The apparatus consists of two open arms (30 × 5 × 0.25 cm), essentially unprotected boards, and two closed arms (30 × 5 × 0.25 cm). These arms emanate from a standard central platform (5 × 5 cm), raised above the floor level to a height of 25 cm. Mice were positioned at the maze’s centre, with the head facing the open arm. The time spent on each arm and the number of entries in each arm were recorded for 5 min [85].

### Assessment of anxiety levels utilising light and dark box

The light/dark transformation test is another behavioral tool used to determine an agent's anxiolytic property. The apparatus consists of a rectangular box (45 × 27 × 27 cm) divided into two compartments, connected by a 7.5 × 7.5 cm opening in the wall. Mice were placed in the illuminated box compartment, and after a 5-min session, the number of entries and time spent in the light and dark box compartments were determined [83].

### Assessment of anxiety levels utilising hole board (HBT)

To determine the anxiolytic properties of the extract on mice using the HBT, we adopted the procedure described by [86]. Each mouse was placed in the centre of the board and was allowed to explore the board freely for 10 min while tracking the number of times the mouse quickly dipped their heads into the holes (active dip). After each test, the board was cleaned with 70% alcohol to remove odour signals [86]**.**

### Assessment of depressive-like behavior using forced swim test (FST)

The forced swim test was also used to assess depressive-like symptoms in experimental animals, following the method described by [83]. In FST, mice were individually forced to swim at a temperature of 25 ± 2 °C for 6 min in a glass jar (height: 20 cm, diameter: 10 cm) filled with water (depth: 15 cm). Immobility duration was recorded during the last 4 min of the 6-min observation period. A mouse was considered immobile when it did not move and held the pangs fixed.

### Assessment of depressive-like behavior using tail suspension test

The tail suspension test (TST) was carried out using an adhesive tape positioned approximately 1 cm from the tip of the mouse’s tail by the protocol described by [85]. The animals were suspended on a retort stand placed 50 cm above the floor. The total immobility period was recorded during the last 4 min of the 6-min test.

### Assessment of depressive-like behavior using the social interaction test

The social interaction test (SIT) was used to assess depressive-like behavior. It consists of a 60 × 40 cm Plexiglas box divided into three chambers (A, B and C) with a small opening (6 × 6 cm), allowing mice to move between chambers. An iron restraining cage was positioned in the two side chambers (A and C). A mouse was placed in the restraining cage in chamber C, while the restraining cage in chamber A was empty. In the centre chamber (chamber B), the test mouse was positioned and allowed to explore the apparatus for 6 min. The cumulative time spent by each mouse in exploring chambers A and C was recorded. At the end of testing, the social preference indexes were defined as follows: (percentage of time spent in the social chamber) (per cent of time spent in the opposite chamber) [87].

### Animal sacrifice and brain tissue collection

Following the behavioral assessments, mice were sacrificed using 0.1 ml/100 kg of sodium pentobarbital intraperitoneally. Experimental animals for histological investigations were perfused transcardially by directly perfusing phosphate buffer and 4% paraformaldehyde through the circulatory system. After about 10 min, the whole brain was harvested and post-fixed in 4% paraformaldehyde until tissue processing.

In addition, the mPFC and hippocampus were harvested and isolated for biochemical assays and homogenised with 1:10 (w/v) or 0.1 mL HCl-butanol in a known volume of ice-cold phosphate-buffered saline (PBS, pH 7.4) and centrifuged for 10 min to obtain a transparent supernatant. The resulting brain homogenates were fixed in ice-cold baths and centrifuged at 3000 rpm for 10 min at 4 °C. The acquired supernatants were immediately stored at − 20 °C before biochemical analyses.

### Preparation of brain tissues for biochemical assays

Analyses of biochemical parameters were carried out on the brain tissue homogenate to assay the effects of *DM* extract on antioxidants and oxidative stress parameters.

### Determination of glutathione

The aliquots of brain supernatant of individual mice in the respective treatment groups were assayed for GSH concentration following Rahman et al. [88]. An equal volume (0.4 ml) of brain homogenate and 20% TCA (0.4 ml) was mixed and then centrifuged using a cold centrifuge at 10,000 rpm at 4 °C for 20 min. 0.25 ml of the supernatant was added to 2 ml of 0.6 mM DTNB, and the final volume was made up to 3 ml with phosphate buffer (0.2 M, pH 8.0). Using a spectrophotometer, the absorbance was then read at 412 nm against a blank reagent. The concentrations of GSH in the brain tissues were expressed as micromoles per gram tissue (μmol/g tissue) [88].

### Determination of superoxide dismutase (SOD) activity

The level of SOD activity in the brain was determined by the methods of Katerji et al. (2019). This method inhibits the auto-oxidative activity of epinephrine at pH 10.2. Superoxide dismutase activity was expressed as units of epinephrine consumed per minute per mg protein [89].

### Estimation of catalase activity

Brain catalase activity was determined according to the protocol adopted by [89]. 2.5 ml phosphate buffer was added to 1.0 ml of aliquots of brain tissue (supernatant) and 2.0 ml of H_2_O_2_. After that, 1 ml of the resulting mixture was pipetted into a test tube, and 2.0 ml of dichromate acetic acid reagent was added. The absorbance of the sample was taken at 240 nm at an interval of 60 s. Catalase activity was determined using this equation: Catalase activity *(*IU*/*L*)* = 0.23 × logAbsorbance 1*/*Absorbance 2/0.00693 [89].

### Estimation of glutathione peroxidase activity (GPx)

The activity of GPx in the brain tissue homogenates was measured using the spectrophotometry method described by [90]. For the reaction mixture, we used 2 mL of brain tissue homogenate, 0.1 mL of 0.01 mol/L 5,5-dithiobis-2- nitrobenzoic acid, 1 mL of 20 mmol/L t-butyl hydroperoxide, and 0.1 mL of 4.8 mmol/L GSH. The reduction in absorbance wavelength at 412 nm was measured with the spectrophotometer [90].

### Estimation of brain nitric oxide level

The concentration of brain nitrite was calculated using a Greiss reagent that serves as an indicator for nitric oxide generation. 100 μL of Greiss reagent was added to 100 μL of the supernatant (1:1 solution of 1% sulfanilamide in 5% phosphoric acid and 0.1% N-1-naphthyl ethylenediamine dihydrochloride), and the absorbance was measured at 540 nm. Nitrite accumulation in the brain (0–100 uM) was estimated by calculating the standard curve [91].

### Estimation of brain level of malondialdehyde (MDA)

MDA, a lipid peroxidation biomarker, was calculated quantitatively by measuring the MDA content using Tsika’s method [92]. In this analysis, the mixture contained 1.0 mL of tissue homogenate,1.0 mL of TCA (10%), and 1.0 mL TBAR (Thiobarbituric acid) (0.67%). Test tubes containing the mixtures were placed in a boiling water bath for 45 min; the resultant mixture was cooled, shifted to an ice bath, and centrifuged 2500 × g for 10 min. The clear supernatant was collected, and the MDA levels were calculated by measuring the absorbance using a spectrophotometer at 532 nm. Values were expressed as nmol MDA per gram of tissue by using a molar extinction coefficient of 1.56 × 105 M^−1^ cm.^−^

### Preparation of brain tissues for histology

After post-fixing brain tissues in 4% paraformaldehyde for 24 h, brain tissues were processed for histological analysis. This involves routine processes, including fixation, dehydration, clearing, and infiltration. Tissues were embedded in molten paraffin wax and sectioned using the rotary microtome. Sections obtained from the hippocampus and medial prefrontal cortex of each treated group were stained using hematoxylin and eosin for general tissue architecture and Golgi stains to visualise neuronal axons and dendrites. The stained tissue images were captured using a computer interface (MagnaFire)-connected Optronics Digital Camera and an Olympus BX-51 Binocular analysis microscope.

### Cell morphology analysis

The diameter, area, length of the axons and dendrites of the neurons in the hippocampus and mPFC were estimated using Image J [93, 94]. A zone of the same diameter and position was drawn to fit the region of interest. All stained cell morphology was estimated from three sections per brain area per mouse, and the average obtained was used for statistical analysis.

### Statistical analysis

Data obtained were analysed using one-way variance analysis (ANOVA). The Bonferroni posthoc test were used to determine the mean significant differences between groups using Graph pad prism (version 5.0, GraphPad Software, La Jolla, CA, USA, GraphPad). The levels of statistical significance were set at *p* ≤ 0.05, while all analysis results were presented as mean ± standard errors (SEM) using bar charts and error bars.

## Data Availability

The datasets used and/or analysed during the current study are available from the corresponding author upon reasonable request.

## Abbreviations

CAT: Catalase
DM: *Datura metel*
EPM: Elevated plus maze
FST: Forced swim test
GPx: Glutathione peroxidase
GSH: Glutathione
mPFC: Medial prefrontal cortex.
MDA: Malondialdehyde
NO: Nitric oxide
NOR: Novel object recognition test
ROS: Reactive oxygen specie
SOD: Sodium dismutase
TST: Tail suspension test
